# Toxicological effect of TiO_2_ nanoparticle-induced myocarditis in mice

**DOI:** 10.1186/s11671-015-1029-6

**Published:** 2015-08-14

**Authors:** Fashui Hong, Ling Wang, Xiaohong Yu, Yingjun Zhou, Jie Hong, Lei Sheng

**Affiliations:** Jiangsu Collaborative Innovation Center of Regional Modern Agriculture & Environmental Protection, Huaiyin Normal University, Huaian, 223300 China; Jiangsu Key Laboratory for Eco-Agricultural Biotechnology around Hongze Lake, Huaiyin Normal University, Huaian, 223300 China; School of Life Sciences, Huaiyin Normal University, Huaian, 223300 China; Library of Soochow University, Suzhou, 215123 China; Medical College of Soochow University, Suzhou, 215123 China

**Keywords:** Titanium dioxide nanoparticles, Heart, Inflammation, Inflammatory cytokines, Transcription factors

## Abstract

Currently, impacts of exposure to TiO_2_ nanoparticles (NPs) on the cardiovascular system are not well understood. The aim of this study was to investigate whether TiO_2_ NPs induce myocarditis and its underlying molecular mechanism in the cardiac inflammation in mice. Mice were exposed to TiO_2_ NPs for 6 months; biochemical parameters of serum and expression of Th1-related and Th2-related cytokines in the heart were investigated. The results showed that TiO_2_ NP exposure resulted in cardiac lesions coupling with pulmonary inflammation; increases of aspartate aminotransferase (AST), creatine kinase (CK), C-reaction protein (CRP), lactate dehydrogenase (LDH), alpha-hydroxybutyrate dehydrogenase (HBDH), adhesion molecule-1 (ICAM-1), and monocyte chemoattractant protein-1 (MCP-1) levels; and a reduction of nitric oxide (NOx) level in the serum. These were associated with increases of nuclear factor-κB (NF-κB), tumor necrosis factor-α (TNF-α), interleukin (IL)-4, IL-6, transforming growth factor-β (TGF-β), creatine kinase, CRP, adhesion molecule-1, and monocyte chemoattractant protein-1, interferon-γ (IFN-γ), signal transducers and activators of transcription (STAT)1, STAT3, or STAT6, GATA-binding domain-3, GATA-binding domain-4, endothelin-1 expression levels, and T-box expressed in T cells expression level that is the master regulator of pro-inflammatory cytokines and transcription factors in the heart. These findings imply that TiO_2_ NP exposure may increase the occurrence and development of cardiovascular diseases.

## Background

Nanotechnology or nanomaterial applications have caused large impacts on human daily life. However, the exposure of workers, consumers, and susceptible groups should be of high concern due to nanomaterial production or uses. Especially, cardiovascular effects due to nanoparticle (NP) exposure may be a possible health risk [1–3]. The mechanisms of these hazardous effects are involved in oxidative stress, inflammation, vasomotor dysfunction, neuronal signaling, and possible translocation of NPs from the airways to the circulation [3]. Inhaled NPs were demonstrated to enter the lungs where they are translocated to the circulatory system, leading to cardiovascular lesions [4]. TiO_2_ NPs are widely applied in an increasing number of products including paints, cosmetics, sunscreen, medicine, food, and toothpaste, and in environmental decontamination, etc. [5–10]. However, previous studies demonstrated that TiO_2_ NP exposure resulted in titanium accumulation in the heart, myocardium dysfunction, oxidative stress, cardiac inflammation, and atherosclerosis in mice [11–13]; increased plaque progression in aorta in mice [14]; and induced endothelial inflammatory response in primary vascular endothelial cells [15]. Other NP exposure, such as ZnO NPs, was also suggested to induce cardiac infarction in rats [16]. However, whether NP exposure is associated with alterations of cytokine response and immune effectors, and imbalance of Th1-related and Th2-related cytokines in cardiovascular damages remains unclear.

As suggested, myocarditis is closely involved in the progression of heart failure due to chronically environmental stimuli such as inhaled particles [5, 7]. Studies showed that inhaled NPs are not reserved in the lung but enter the blood circulation and distribute to distant organs including the liver, spleen, kidneys, lungs, and heart [17–19]. Therefore, we hypothesized that NP exposure may give rise to various venenous stimuli that cause secretion of both leukocyte soluble adhesion molecules, facilitating the attachment of monocytes to endothelial cells, and chemokines, thus resulting in the monocytes’ migration into the subintimal space. The transformation of monocytes into macrophages led to myocarditis in animals and humans. The pathophysiological changes provide potential targets for identifying and monitoring the NP-induced inflammatory process, while potential targets are involved in pro-inflammatory risk factors such as pro-inflammatory cytokines, adhesion molecules, and inflammatory stimuli [20]. Thus, it is necessary to confirm the mechanism of NP-induced myocarditis.

In this study, therefore, myocardium parameters and alterations in the inflammatory cytokines and transcription factor expression in mouse heart were investigated to determine whether TiO_2_ NP-induced cardiac lesion is mediated by Th1-related and Th2-related cytokines in mice.

## Methods

### Chemicals

For the preparation, characteristics of anatase TiO_2_ NPs have been described in our previous work [21–23]. Hydroxypropylmethylcellulose (HPMC) 0.5 % *w*/*v* was employed as an agent for suspending diffusion. TiO_2_ powder was dispersed onto the surface of 0.5 % *w*/*v* HPMC solution, and then the suspending solutions containing TiO_2_ particles were treated ultrasonically for 15–20 min and mechanically vibrated for 2 or 3 min [22, 23]. The particle sizes of NPs suspended in 0.5 % *w*/*v* HPMC solution following incubation (5 mg/L) were determined using a TecnaiG220 transmission electron microscope (TEM) (FEI Co., USA) operating at 100 kV, respectively. The surface area of sample was detected by Brunauer–Emmett–Teller (BET) adsorption measurements on a Micromeritics ASAP 2020M+ C instrument (Micromeritics Co., USA). The average aggregate or agglomerate size of the TiO_2_ NPs in 0.5 % (*w*/*v*) HPMC solution (5 mg/mL) was determined by dynamic light scattering (DLS) using a Zeta PALS + BI-90 Plus (Brookhaven Instruments Corp., USA) at a wavelength of 659 nm [22, 23]. The characteristics were about 5.5 nm for average particle size, 174.8 m^2^/g for the surface area, mainly 294 nm for the mean hydrodynamic diameter, and 9.28 mV for the *ζ* potential [21–23].

### Ethics Statement

All experiments were approved by the Animal Experimental Committee of Soochow University (Grant 2111270) and in accordance with the National Institutes of Health Guidelines for the Care and Use of Laboratory Animals (NIH Guidelines).

### Animals and Treatment

One hundred sixty 4-week-old CD-1 (ICR) male mice (20 ± 2 g body weight) were purchased from the Animal Center of Soochow University (China). Immediately after arrival, all mice were weighed and randomly allocated into four subgroups (*n* = 40), including a control group treated with 0.5 % *w*/*v* HPMC and three experimental groups treated with 1.25, 2.5, and 5 mg/kg TiO_2_ NPs [24], respectively. For dose selection, we consulted a report of the World Health Organization from 1969. According to the report, the LD 50 of TiO_2_ for rats is >12 g/kg body weight after oral administration. We also consulted that in November 2005, the United States National Institute for Occupational Safety and Health (NIOSH) proposed a recommended exposure limit (REL) for TiO_2_ NPs at 0.3 mg/m^3^ (NIOSH). In Japan, the acceptable exposure concentration of TiO_2_ NPs was estimated to be 1.2 mg/m^3^ as a time weighted average (TWA) for an 8-h workday and a 40-h workweek [25, 26]. In Europe, food-grade TiO_2_ is approximately 36 % of the TiO_2_ NPs that are smaller than 100 nm in at least one dimension, this exposure limit decreases to approximately 0.1 mg TiO_2_/person/day of nanoscale TiO_2_ [27]. Mice were housed in cages and were kept under specific pathogen-free (SPF) conditions. Room environment was set up at 24 ± 2 °C with 60 ± 10 % of relative humidity and a 12-h light/dark cycle. Distilled water and sterilized food for mice were available *ad libitum*. They were acclimatized and quarantined to this environment for 5 days prior to dosing.

Before the nasal instillation to the mice, TiO_2_ NP powder was dispersed onto the surface of 0.5 % *w*/*v* HPMC and re-suspended TiO_2_ NPs were homogenized by a sonicator for 30 min and mechanically vibrated for 5 min. The volume of TiO_2_ NP suspensions was calculated for each mouse after weighing mice and was administered to the mice by nasal administration every other day for 6 months. After the final exposure to TiO_2_ NPs (e.g., 24 h following the last exposure), all the mice were sacrificed after anesthetization with ether. Blood sera were collected and stored at −20 °C before use. Every effort was made to minimize animal suffering in each experiment. All experiments were performed in accordance with the Guiding Principles in the Use of Animals in Toxicology.

### Assay of Pulmonary Inflammation

After blood collection, the lungs from the control and TiO_2_ NP-treated groups were immediately lavaged twice with phosphate buffer saline (PBS). An average of >90 % of the total instilled PBS volume was retrieved both times, and the amounts did not differ among the groups. The resulting fluid was centrifuged at 400×*g* for 10 min at 4 °C to separate the cells from the supernatant containing various surfactants and enzymes. The cell pellet was used for enumeration of total and differential cell counts as described by AshaRani et al [28]. Macrophages, lymphocytes, neutrophils, and eosinophils recovered from the bronchoalveolar lavage fluid (BALF) were counted using dark field microscopy to examine the extent of phagocytosis. The inflammatory cytokines interleukin-6 (IL-6) and tumor necrosis factor alpha (TNF-α) were detected in the primary cell-free BALF by ELISA commercial kits (R&D Systems, Minneapolis, MN, USA).

### Histopathological Examination of Lung and Heart

Lungs or hearts were fixed with 10 % neutral buffered formalin for 3 days, which were embedded in paraffin blocks, sliced to 5-μm thickness, placed on separate glass slides, and were stained with hematoxylin and eosin (H&E). After H&E staining, the sections were evaluated by blinding test, using an optical microscope (U-III Multi-point Sensor System; Nikon, Tokyo, Japan).

### Biochemical Assay of Myocardium Function

In the present study, the activities of aminotransferase (AST), creatine kinase (CK), cross-reaction protein (CRP), lactate dehydrogenase (LDH), and alpha-hydroxybutyrate dehydrogenase (HBDH) in the serum were determined using commercial assay kits (Nanjing Jiancheng Bioengineering Institute, Jiangsu, China) according to the manufacturer’s instructions. Levels of eotaxin (ET)-1, total nitric oxide (nitrite + nitrate, NOx), intercellular adhesion molecule-1 (ICAM-1), and monocyte chemoattractant protein-1 (MCP-1) in the serum was assayed for evaluating myocardium function using commercial kits (R&D Systems, Minneapolis, MN).

### Assay of Cytokine Expression

Total RNA was extracted from individual heart using Tripure Isolation Reagent (Roche, USA) according to the manufacturer’s instructions. Probes and cycling condition were optimized in accordance with MIQE guidelines for PCR [29]. cDNA was used for the real-time PCR by employing primers designed using Primer Express Software according to the software guidelines. PCR primers used in the gene expression analysis are listed in Table 1. Gene expression levels were calculated as a ratio to the expression of the reference gene, GAPDH, and data were analyzed using the ΔΔCt method. The probes for *NF-κB*, *IκB*, *TNF-α*, *IL-1β*, *IL-4*, *IL-6*, *CRP*, *CK*, *TGF-β*, *IFN-γ*, *VCAM-1*, *MCP-1*, *STAT1, STAT3, STAT6, GATA3*, *GATA4*, *T-bet,* and *VEGF* were designed by the manufacturer and purchased from Shinegene Company (Shanghai, China). The RT-qPCR data were processed with the sequence detection software version 1.3.1 following the method of Schefe et al. [30].

**Table 1:** Real-time PCR primer pairs. PCR primers used in the gene expression analysis

Gene name	Description	Primer sequence	Primer size (bp)
Refer-GAPDH	mGAPDH-F	5′-TGTGTCCGTCGTGGATCTGA-3′	
	mGAPDH-R	5′-TTGCTGTTGAAGTCGCAGGAG-3′	150
*CK*	mck-F	5′-GAGATCTTCAAGAAGGCTGGTCA-3′	
	mck-R	5′-GAGATGTCGAACACGGCG-3′	227
*CRP*	mcrp-F	5′-GCGGAAAAGTCTG-CACAAGG-3	
	mcrp-R	5′-GGAGATAGCACAAAGTCCCACAT-3	153
*ET-1*	mET-1-F	5′-AGACCACAGACCAAGGGAACA-3′	
	mET-1-R	5′-TCTGCTTGGCAGAAATTCCA-3′	392
*ICAM-1*	mICAM-1-F	5′-AGACACAAGCAAGAGAAGAAAAGG-3′	
	mICAM-1-R	5′-TTGGGAACAAAGGTAGGAATGTAT-3′	425
*MCP-1*	mMCP-1-F	5′-GCTGACCCCAAGAAGGAATG-3′	
	mMCP-1-R	5′-TTGAGGTGGTTGTGGAAAAGG-3′	184
*NF-κB*	mNF-κB-F	5′-GTGGAGGCATGTTCGGTAGTG-3′	
	mN-κB-R	5′-TCTTGGCACAATCTTTAGGGC-3′	195
*IκB*	mIκB-F	5’-GGTGCAGGAGTGTTGGTGG-3′	
	mIκB-R	5′-TGGCTGGTGTCTGGGGTAC-3′	173
*IL-1β*	m IL-1β-F	5′-GCTTCAGGCAGGCAGTATCA-3′	
	mIL-1β-R	5′-TGCAGTTGTCTAATGGGAACG-3′	196
*TNF-α*	mTNF-α-F	5′-CCCTCCAGAAAAGACACCATG-3′	
	mTNF-α-R	5′-CACCCCGAAGTTCAGTAGACAG-3′	183
*TGF-β*	mCcl21a-F	5′-CACGGTCCAACTCACAGGC-3′	
	mCcl21a-R	5′-TTGAAGCAGGGCAAGGGT-3′	102
*IL-4*	mIL-4-F	5′-TGTAGGGCTTCCAAGGTGCT-3′	
	mIL-4-R	5′-TGATGCTCTTTAGGCTTTCCAG-3′	199
*IL-6*	mIL-6-F	5′-GGGACTGATGCTGGTGACAAC-3′	
	mIL-6-R	5′-CAACTCTTTTCTCATTTCCACGA-3′	163
*STAT1*	mSTAT1-F	5′-ACGCTGCCTATGATGTCTCG-3′	
	mSTAT1-R	5′-ACGGGATCTTCTTGGAAGTTATC-3′	163
*STAT3*	mSTAT3-F	5′-TGACCAATAACCCCAAGAACG-3′	
	mSTAT3-R	5′-TGACACCCTGAGTAGTTCACACC-3′	181
*STAT6*	mSTAT6-F	5′-AGCATCTTGCCGCACATCA-3′	
	mSTAT6-R	5′-GGCAGGTGGCGGAACTCT-3′	128
*GATA3*	mGATA3-F	5′-CCACGGGAGCCAGGTATG-3′	
	mGATA3-R	5′-CGGAGGGTAAACGGACAGAG-3′	169
*GATA 4*	mGata4-F	5′-CCTGGAAGACACCCCAATCT-3′	
	mGata4-R	5′-GGTAGTGTCCCGTCCCATCT-3′	115
*T-bet*	mT-bet-F	5′-TGGACCCAACTGTCAACTGC-3′	
	mT-bet-R	5′-CTCGGAACTCCGCTTCATAAC-3′	173

To determine protein levels of nuclear factor-κB (NF-κB), IκB, TNF-α, interleukin (IL)-1β, IL-4, and IL-6, CRP, CK, transforming growth factor-β (TGF-β), interferon-γ (IFN-γ), ICAM-1, MCP-1, signal transducers and activators of transcription factor (STAT)1, STAT3, and STAT6, GATA3, GATA4, ET-1, T-box expressed in T cell (T-bet), and vascular endothelial growth factor (VEGF) in the heart (*n* = 5 each), total protein from the frozen heart tissues (*n* = 5 in each group) from experimental and control mice was extracted using Cell Lysis Kits (GENMED SCIENTIFICS INC.USA) and quantified using BCA protein assay kits (GENMED SCIENTIFICS INC.USA). ELISA was performed using commercial kits that were selective for each respective protein (R&D Systems, USA), following the manufacturer’s instructions.

### Statistical Analysis

Data were represented as mean ± standard deviation (SD). Statistical analyses were performed by SPSS 19.0 software (Chicago, IL, USA), and statistical comparisons were analyzed using one-way ANOVA followed by Tukey’s HSD post hoc test. Differences were considered statistically significant when the *P* value was less than 0.05.

## Results

### Pulmonary or Heart Inflammation

**Fig. 1:**
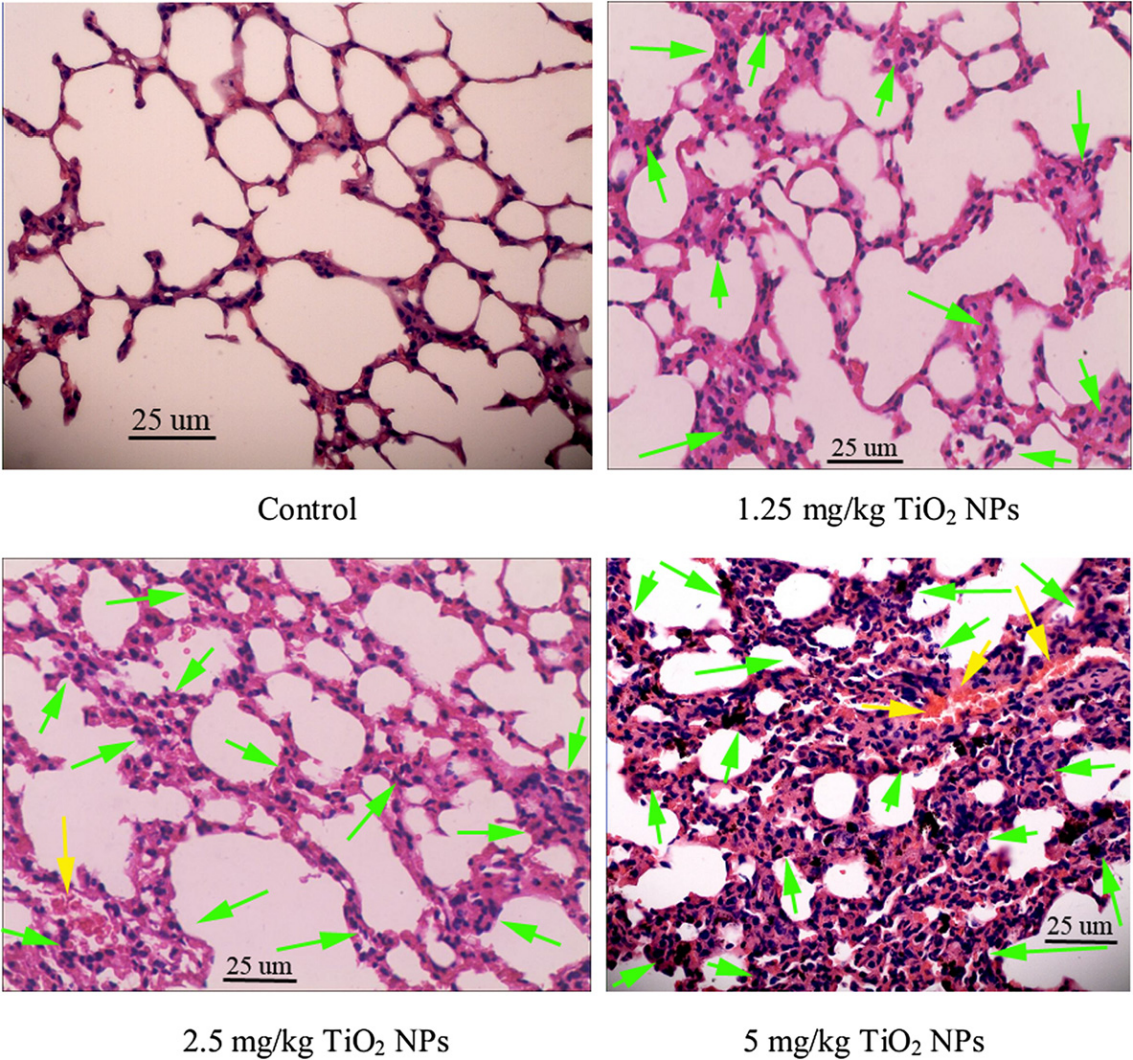
Histopathological observation of lungs of mice after nasal administration of TiO_2_ NPs for 6 months (*n* = 5). TiO_2_ NP-exposed mice show infiltration of inflammatory cells (*green arrow*) and bleeding (*yellow arrow*) in the lung

**Fig. 2:**
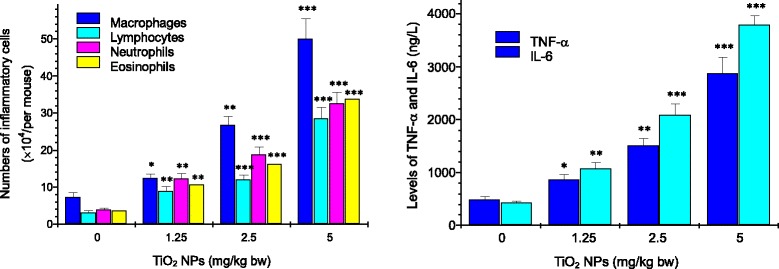
Numbers of inflammatory cells and levels of TNF-α and IL-6 in BALF. *P < 0.05, **P < 0.01, and ***P < 0.001. Values represent means ± SD (*n* = 5)

Figure 1 exhibits thickening of the alveolar septae, bleeding, and infiltration of inflammatory cells in the TiO_2_ NP-treated mouse lungs. In addition, significant black agglomerates were observed in the lung samples exposed to 5 mg/kg of TiO_2_ NPs (Fig. 1). Confocal Raman microscopy further suggested that the black agglomerate was due to the deposition of TiO_2_ NPs in the lungs [31]. With increasing TiO_2_ NP dose, the numbers of inflammatory cells such as macrophages, lymphocytes, neutrophils, and eosinophils and the levels of inflammatory cytokines such as IL-6 and TNF-α in the BALF were greatly elevated as compared to the control (Fig. 2, *P* < 0.05).

**Fig. 3:**
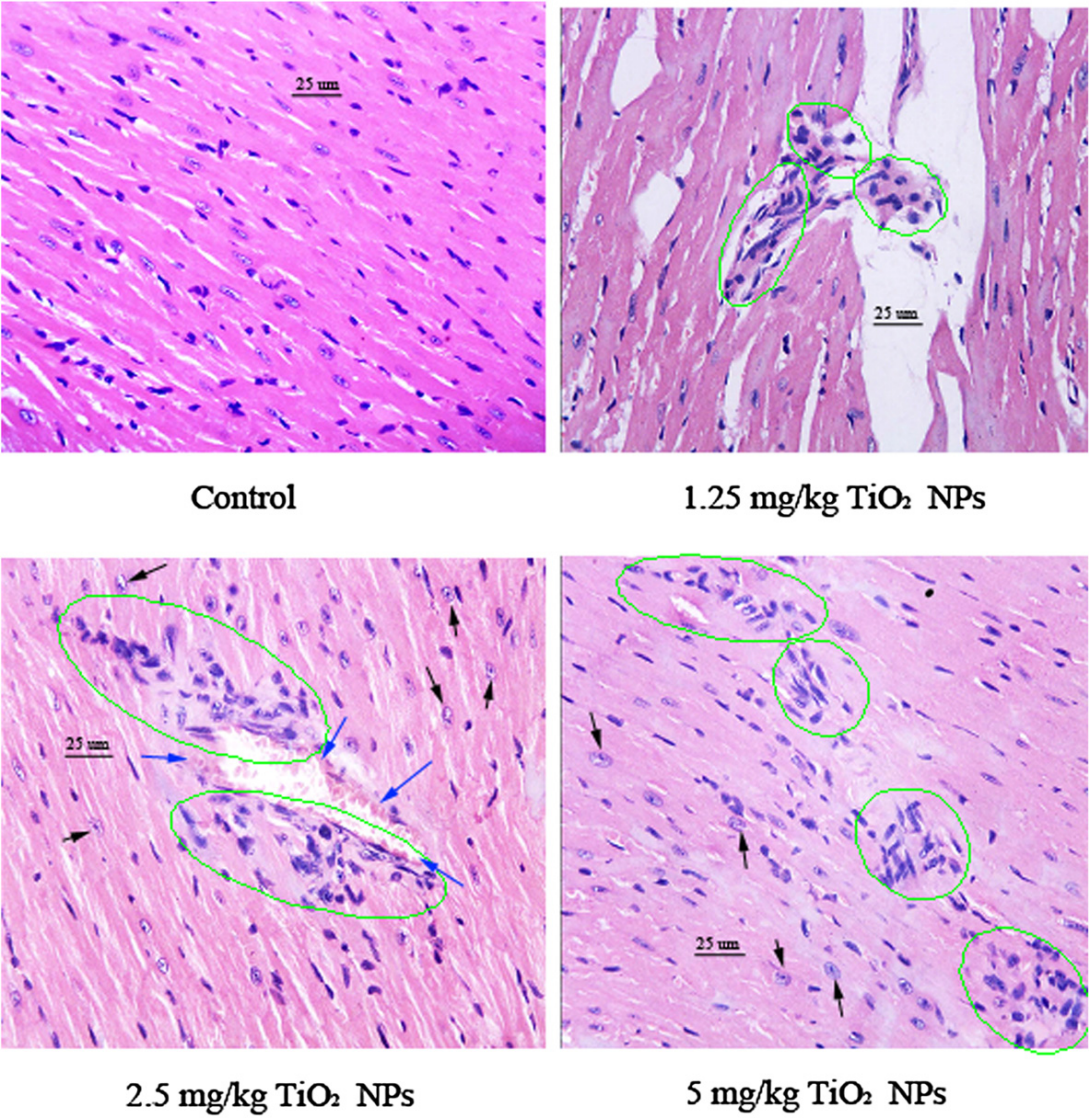
Histopathological observation of hearts after nasal administration of TiO_2_ NPs for 6 months (*n* = 5). *Black arrow* indicates myocardial cells swelling; *green circle* indicates infiltration of inflammatory cells; *blue arrow* indicates hemolysis or bleeding

The histological examinations of the heart sections are shown in Fig. 3. Unexposed heart samples exhibited normal architecture (Fig. 3), whereas those from mice exposed to increasing TiO_2_ NP dose presented severe pathological changes, including infiltration of inflammatory cells, myocardial cells swelling, sparse cardiac muscle fibers, and disorder of muscle cell array (Fig. 3).

### Biochemical Parameters

**Fig. 4:**
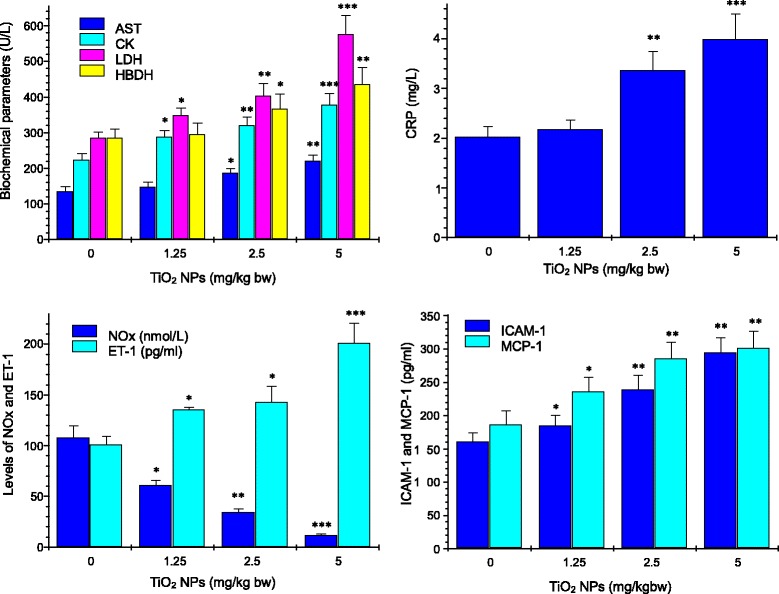
Effect of TiO_2_ NPs on biochemical parameters in the serum of myocardium after nasal administration with TiO_2_ NPs for 6 months. *P < 0.05, **P < 0.01, and ***P < 0.001. Values represent means ± SD (*n* = 5)

The changes of biochemical parameters in the serum induced by TiO_2_ NP exposure are presented in Fig. 4. With increasing TiO_2_ NP dose, inflammatory parameters, including AST, CK, CRP, LDH, HBDH, ICAM-1, and MCP-1, increased gradually (*P* < 0.05). These results indicated that chronic TiO_2_ NP exposure made serious cardiac inflammation of mice. ET-1 and NOx were determined in mice to evaluate vascular endothelial function after TiO_2_ NP exposure. Figure 4 lists the levels of ET-1 and NOx in the serum, showing an increase of ET-1 level (*P* < 0.05) and a decrease of NOx level (*P* < 0.05). It indicated that TiO_2_ NP-exposed mice had an endothelial dysfunction.

### Expression of Th1 and Th2 Cytokine mRNA and Proteins

**Fig. 5:**
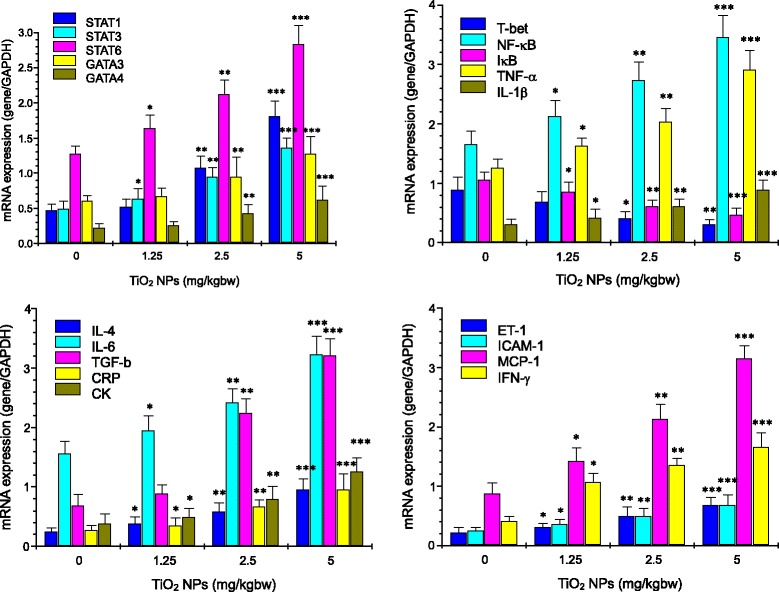
Effect of TiO_2_ NPs on mRNA expression of myocarditis-related genes in mouse heart by real-time PCR analysis. *P < 0.05, **P < 0.01, and ***P < 0.001. Values represent means ± SD (*n* = 5)

**Fig. 6:**
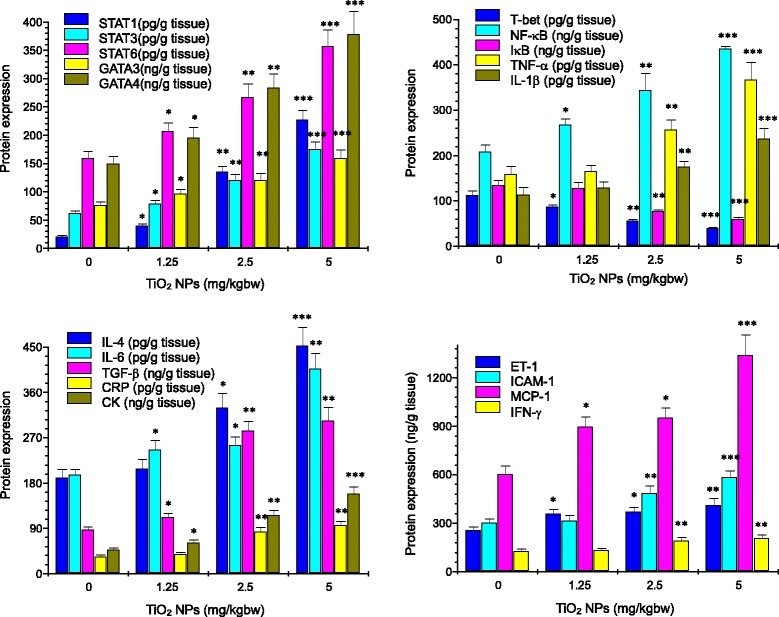
Effect of TiO_2_ NPs on protein expression of myocarditis-related genes in mouse heart by ELISA analysis. *P < 0.05, **P < 0.01, and ***P < 0.001. Values represent means ± SD (*n* = 5)

To further confirm whether TiO_2_ NP exposure resulted in imbalance of Th1-related and Th2-related cytokines, including NF-κB, TNF-α, IL-1β, IL-4, IL-6, CK, CRP, ET-1, TGF-β, IFN-γ, ICAM-1, MCP-1, STAT1, STAT3, STAT6, T-bet, GATA3, and GATA4, in the TiO_2_ NP-induced cardiac injury, changes of the cytokine gene and protein expression in mouse heart were examined and are showed in Figs. 5 and 6. Mice with TiO_2_-NP-induced cardiac damages presented with a significant, dose-dependent reduction in the nuclear IκB expression and a dose-dependent marked increase in expression of these genes and proteins mentioned above in the cardiac tissue (Figs. 5 and 6, *P* < 0.05). These findings pointed to the imbalance of Th1-related and Th2-related cytokines in mice following exposure to TiO_2_ NPs.

## Discussion

Manufactured NPs have been suggested to increase the risk and incidence of cardiovascular diseases such as myocarditis [5, 17, 18, 31, 32]. Occupational and environmental exposure of TiO_2_ NPs may be one of risk factors for increased myocarditis in humans. Air dust containing TiO_2_ NPs may result in higher inhalation absorption and subsequent translocation of TiO_2_ NPs into the circulatory system. Seaton et al. indicated that inhaled particles act as their cardiovascular effects indirectly through the passage of inflammatory mediators from the lung to the systemic circulation [33]. In the present study, TiO_2_ NP exposure led to a severe pulmonary inflammation characterized by infiltration of macrophages, lymphocytes, neutrophils, and eosinophils into the airways (Figs. 1 and 2), especially, there was a close association with level of pulmonary inflammation and the cardiac damages such as myocarditis and myocardial cell swelling (Fig. 3). Levels of TNF-α and IL-6 proteins in the BALF were elevated due to TiO_2_ NP exposure (Fig. 2), which were associated with pulmonary inflammation (Figs. 1 and 2). It implies that the inflammatory pathways may be likely to contribute to the cardiovascular effects of TiO_2_ NPs, neither pulmonary nor systemic inflammation alone can account for the myocarditis actions of TiO_2_ NPs. To confirm mechanism of the cardiac lesions of mice following exposure to TiO_2_ NPs, in this study, we examined alterations of different serum parameters and expression of Th1-related and Th2-related cytokines, and the results are discussed as follows.

The cardiac lesions due to TiO_2_ NP exposure were reflected to severe myocardium biochemical dysfunction, marked by significant increases of AST, CK, LDH, CRP, HBDH, ICAM-1, and MCP-1 levels, and NOx reduction in the serum (Fig. 4). Our previous study has also indicated that intragastric administration of TiO_2_ NPs for 90 days resulted in increased CK activity and severe pathological changes of heart in mice such as inflammation [12]. Elevated levels of biomarkers involving systemic inflammation, immune function, and ventricular remodeling, including AST, LDH, CRP, CK, HBDH, TNF-α, ET-1, ICAM-1, and MCP-1, also have been related to morbidity and mortality among heart failure patients [34–37]. The expression of adhesion molecules such as MCP-1 and ICAM-1 is associated with early atherosclerotic formation [38]. Importantly, increased ICAM-1 expression exacerbated the inflammatory process via facilitating leukocyte adhesion to the endothelium and releasing activated leukocytes to the inflammatory sites [39]. In our study, increased levels of AST, LDH, CRP, CK, HBDH, TNF-α, ICAM-1, and MCP-1 in the serum and tissue mRNA and protein expression by TiO_2_ NP exposure may be associated with inflammatory responses in the heart. Increased AST and CRP were demonstrated to be closely involved in the liver lesions of mice due to TiO_2_ NP exposure [13, 23].

As suggested and mentioned above, endothelial dysfunction after exposure to TiO_2_ NPs may be related to susceptibility of mice. In the present study, our data showed that the NOx level in the serum in the TiO_2_ NP-exposed mice was significantly decreased (Fig. 4), whereas ET-1 concentration in the serum and ET-1 expression in the heart were markedly elevated (Figs. 4, 5, and 6). It is well known that NO is very important for endothelial function and the dysfunction exacerbated cardiovascular lesions [40]. In addition, NO is also demonstrated to decrease inflammation [41] and platelet adhesion [42, 43]. Excessive endothelium-restricted ET-1 expression in mice can not only cause endothelial dysfunction but also impair NOx-dependent vasorelaxation in resistance vessels and intensify vascular reactive oxygen species (ROS) production [44]. Our findings showed that TiO_2_ NP-exposed mice exhibited severe inflammation and vascular endothelial dysfunction, implying that the lesions may be involved in NOx reduction and ET-1 overexpression due to TiO_2_ NP exposure.

TGF-β can promote the synthesis of various cytokines and growth factors that are involved in the formation of cardiac fibrosis and mediate the transition from acute inflammation to fibrosis in ischemic heart disease [45]. Resolution of inflammation and progressive remodeling are suggested to be involved in TGF-β overexpression in the myocardium [46–51]. In our study, therefore, TGF-β expression was analyzed by RT-PCR and ELISA, showing that TiO_2_ NP exposure significantly upregulated expression of TGF-β in mouse heart (Figs. 5 and 6) coupling with myocardial cell swollen and increased inflammatory cells in mice (Fig. 3). It is likely that TiO_2_ NP-induced hypertrophic myocardium may involve in TGF-β overexpression in mice.

Myocarditis is suggested to be a T cell-mediated autoimmune disease. Activated T cells can release numerous chemokines and cytokines, recruiting and activating other inflammatory cells (such as macrophages, neutrophils, and mast cells) [52]. Overexpression of cytokines induced by inflammatory stimuli exacerbates the progression of myocardial damage in patients with myocarditis [51]. Immunological and pathophysiological events remarkably contribute to increase mast cells [53], resulting in cardiac inflammation and fibrosis [54]. IL-1 is considered to play a critical role and is highly expressed in hearts with myocarditis [55]. Therefore, it is important to decrease myocarditis via decreasing expression of inflammatory cytokines (such as IL-1β and TNF-α) and a master transcriptional factor NF-κB that can modulate many genes responsible for both the innate and adaptive immune response. In the present study, TiO_2_ NP exposure resulted in myocardial cells swelling and infiltration of inflammatory cells in mouse heart (Fig. 3), which were associated with increased expression of NF-κB, IL-1β, and TNF-α in the TiO_2_ NP-exposed heart (Figs. 5 and 6). Furthermore, the gene and protein expression of Th1- and Th2-related cytokines including IL-4, IL-6, and IFN-γ was significantly increased in the TiO_2_ NP-exposed mouse heart (Figs. 5 and 6). Additionally, TiO_2_ NP exposure induced marked upregulation of Th2-related transcription factors including STAT1, STAT3, STAT6, GATA3, and GATA4, and Th1-related transcription factors such as T-bet in the heart (Figs. 5 and 6). As known, the immune system is closely associated with the progression of inflammation of the cardiovascular system. In regulating immune function and inflammatory response in the cardiovascular system, the balance between Th1-related cytokine expression and Th2-related cytokine expression is demonstrated to be important [56–58].

As suggested, STAT1 is associated with IFN-γ expression and plays an important role in Th1-specific cytokine expression [59]. IFN-γ can induce Th1 activation by activating STAT1, which in turn activates T-bet. The upregulated T-bet expression is closely related to the mediation of STAT1. Wei et al. showed that the STAT1-, STAT3-, STAT6-dependent pathways mediated the activation of T-bet, GATA3, and GATA4 [60], suggesting that the upregulation of STAT1, STAT3, and STAT6 may be risk factors for the progression of cardiovascular injuries following exposure to TiO_2_ NPs. Furthermore, GATA3, GAT4, and STAT6 were suggested to influence Th2 cell differentiation and induce expression of IL-4 and IL-6 [61, 62]. GATA3 and GATA4 can drive differentiation of Th2 cells, whereas T-bet can promote expansion of Th1 cells [63]. Wojakowski et al. observed that GATA-4 expression in patients with acute myocardial infarction related to the increased levels of inflammatory cytokines was significantly upregulated [64]. The balance between GATAs/T-bet may be related to the fate of T cell polarization during the immune response. Szabo et al. demonstrated that T-bet can drive chromatin remodeling of the IFN-γ locus and plays a key role in Th1 cell development and regulation [65]. Increased level of STAT1, STAT3, STAT6, GATA3, GATA4, IL-4, IL-6, IFN-γ, and T-bet expression (Figs. 5 and 6) demonstrated that TiO_2_ NP exposure could alter the expression of Th1- and Th2-related transcription factors, suggesting that chronic exposure to TiO_2_ NPs impaired the balance of Th1 and Th2 at the transcriptional levels. Because the Th1/Th2 imbalance could promote the progression of allergy or infection [66], our results partly explained that TiO_2_ NPs may impair balance of Th1 and Th2 cytokines and alter the immune response toward the allergy-related Th2 cytokines. IL-6 is a vital procoagulant cytokine, and it contributes to enhance plasma CRP concentration, which exacerbates inflammatory and procoagulant responses [67]. Inflammatory cytokines, including IL-1β, TNF-α, and CRP, have been suggested to induce the expression of cellular adhesion molecules, which promote adhesion of leukocytes to the vascular endothelium [68, 69]. CRP can also activate monocytes to express a glycoprotein tissue factor that plays a critical role in coagulation [70]. Importantly, endothelium-derived NOx production is decreased at the damaged vascular site. Therefore, a reduction in NOx activity exacerbates a pro-inflammatory and pro-thrombotic milieu. CRP may itself play an important role in decreasing NOx production and bioavailability [71]. Therefore, cardiac lesion caused by TiO_2_ NP exposure may be associated with alterations of cytokine expression and immunological function in myocardium and the imbalance of Th1 and Th2 cytokines.

## Conclusions

TiO_2_ NP exposure resulted in cardiac inflammation coupling with pulmonary inflammation, which may be associated with immune dysfunction and imbalance of Th1-related cytokine expression and Th2-related cytokine expression in mouse heart. The finding exhibits new insight into the mechanisms of the TiO_2_ NP-induced cardiovascular damage. However, the interaction of other Th1/Th2-related cytokines associated with TiO_2_ NP-induced cardiovascular injury will be further investigated in future.
